# Securing UAV Flying Ad Hoc Wireless Networks: Authentication Development for Robust Communications †

**DOI:** 10.3390/s25041194

**Published:** 2025-02-15

**Authors:** Muhammet A. Sen, Saba Al-Rubaye, Antonios Tsourdos

**Affiliations:** School of Aerospace, Transportation and Manufacturing, Cranfield University, Cranfield MK43 0AL, UK; s.alrubaye@cranfield.ac.uk (S.A.-R.); antonios.tsourdos@cranfield.ac.uk (A.T.)

**Keywords:** unmanned aerial vehicles, authentication protocol, physical unclonable function

## Abstract

Unmanned Aerial Vehicles (UAVs) have revolutionized numerous domains by introducing exceptional capabilities and efficiencies. As UAVs become increasingly integrated into critical operations, ensuring the security of their communication channels emerges as a paramount concern. This paper investigates the importance of safeguarding UAV communication against cyber threats, considering both intra-UAV and UAV–ground station interactions in the scope of the Flying Ad Hoc Networks (FANETs). To leverage the advancements in security methodologies, particularly focusing on Physical Unclonable Functions (PUFs), this paper proposes a novel authentication framework tailored for UAV networking systems. Investigating the existing literature, we categorize related studies into authentication strategies, illuminating the evolving landscape of UAV security. The proposed framework demonstrated a high level of security with lower communication and computation costs in comparison with selected studies with similar types of attacks. This paper highlights the urgent need for strong security measures to mitigate the increasing threats that UAVs encounter and ensure their sustained effectiveness in a variety of applications. The results indicate that the proposed protocol is sufficiently secure and, in terms of communication cost, achieves an 18% improvement compared to the best protocol in the referenced studies.

## 1. Introduction

Unmanned Aerial Vehicles (UAVs) and their effective use have changed the balance in the field and started a new era. A single UAV or a fleet of UAVs can frequently undertake important missions, particularly in combat situations. During these critical operations, security of communication and data is crucial. Untrusted communication environments can lead to the misdirection of crucial payload carried by UAVs or the disclosure of important tactical data. Conventional authentication methods for communication security include ID-based, certificateless, and public key infrastructure-based authentication. These systems are still vulnerable to certain cyber attacks, and some of them might bring computational complexity, which is inappropriate for industrial drones [1].The development of UAVs has caused a paradigm change in a number of industries in recent years, bringing about novel capabilities and efficiencies that were not possible before. UAVs have become crucial instruments in significant operations, beyond traditional operational boundaries in areas such as disaster relief, agricultural monitoring, and military reconnaissance [2]. However, as their utilization continues to expand, it becomes even more important to protect their communication channels against evolving cyber threats. Although operating at high altitudes makes them less accessible to outside interference, the growing sophistication and accessibility of cyber attack tools make these UAVs vulnerable to severe security threats [3,4].

Ensuring the security of UAV communication channels is vital, as even minor breaches can have significant operational consequences. In crucial operations, the smallest breach in communication integrity can have significant consequences, since real-time data transfer and command execution are essential. Threats ranging from signal interference and data manipulation to unauthorized access pose significant risks to the reliability, confidentiality, and operational efficacy of UAV systems. To address these challenges, various security methodologies have been proposed by researchers, including encryption, secure communication protocols, and blockchain-based systems [5,6]. Among these approaches, Physical Unclonable Functions have emerged as a promising solution for authenticating UAV communication channels.

### 1.1. Physical Unclonable Functions

**Figure 1:**
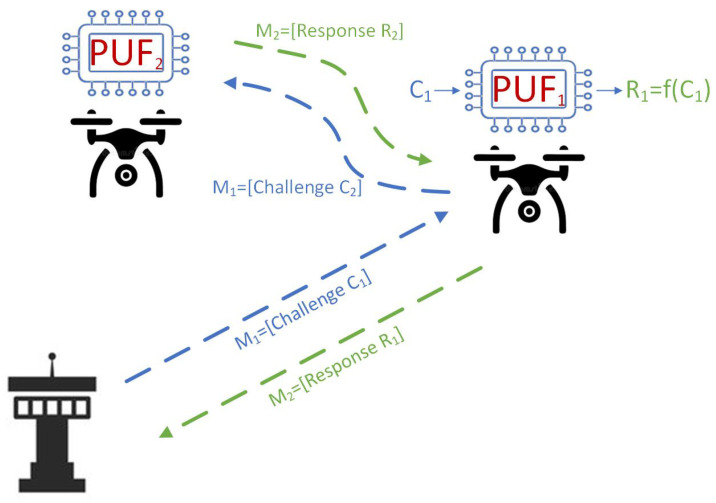
PUFs in UAV authentication: fundamental operational mechanism.

PUFs provide strong defense against cloning and tampering attacks by creating unique identities using the physical differences that naturally exist in hardware components. Figure 1 illustrates the fundamental operational mechanism of PUFs in UAV authentication. It demonstrates how physical characteristics inherent to each UAV can be used to produce a unique and secure identifier, which plays a crucial role in the authentication process.

**Figure 2:**
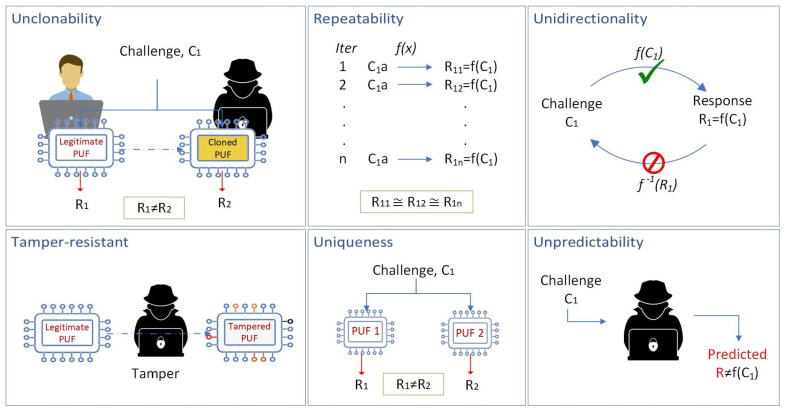
Characteristics of PUFs.

The essential characteristics of PUFs that contribute to their effectiveness in security applications include unclonability, repeatability, unidirectionality, tamper resistance, uniqueness, and unpredictability. PUFs cannot be cloned because the physical variations that produce their responses are unique to each PUF, making replication nearly impossible. They also exhibit repeatability, meaning that despite their unpredictability, a PUF will produce the same response to a given challenge under consistent conditions, ensuring reliable authentication. Additionally, PUFs are unidirectional, making it computationally infeasible to derive the challenge from the response, which protects against reverse-engineering attempts. Their tamper resistance is another crucial feature; any physical alteration to a PUF will change its response characteristics, thereby invalidating its responses and preventing cloning or tampering. Furthermore, the uniqueness of PUFs ensures that each one has distinct physical properties, resulting in unique responses that can differentiate between different UAVs. Lastly, the unpredictability of PUF responses ensures that even if an attacker knows the challenge, they cannot predict the response, adding an additional layer of security. Figure 2 provides an overview of PUF characteristics, emphasizing the robustness of PUFs in providing secure, unique, and tamper-resistant identifiers for UAV authentication.

By incorporating PUFs into the authentication protocol, UAV communication channels can be secured against a wide range of cyber threats, including cloning, replay attacks, and tampering. The unique responses generated by PUFs provide a robust method for verifying the identity of UAVs, ensuring that only legitimate devices can participate in the network. This enhances the overall security and reliability of UAV operations, particularly in critical applications where data integrity and confidentiality are important.

### 1.2. Flying Ad Hoc Networks

**Figure 3:**
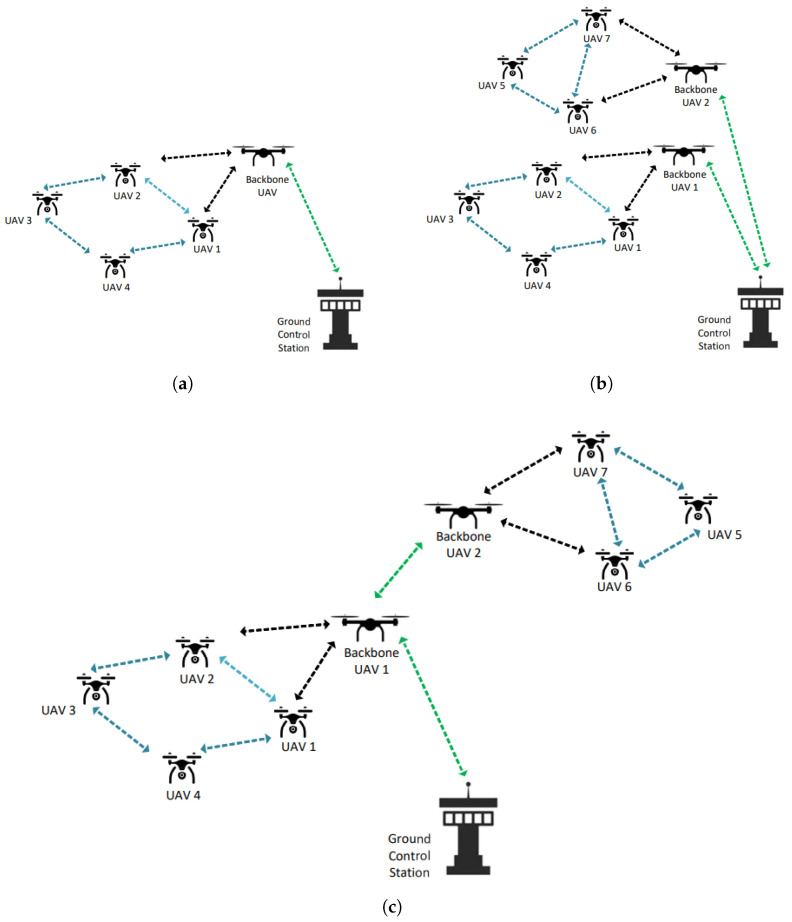
FANET Architectures. (**a**) Basic FANET model, (**b**) Multi-Group FANET, (**c**) Multi-Layer FANET.

FANET is an advanced model of the Vehicular Ad Hoc Network (VANET), offering features such as lower cost [7] in UAV swarms, higher scalability [8], increased survivability [9], reduced mission time [10], and less detection on radar [11]. Emerging as a 3D network to meet these needs, FANET plays a crucial role in enhancing the interoperability of UAVs during flight. In a FANET operation, UAVs collaborate with each other to expand their operational area, maintain continuous information exchange, and prevent collisions. Mission-related information is distributed by a backbone UAV and sequentially transferred among the UAVs. This collective of multiple UAVs working together is also known as a swarm. UAVs joining and leaving the swarm constantly update the backbone UAV through neighboring UAVs, ensuring proper authorization and coordination. Establishing the network among UAVs in FANET is possible via a variety of methods [12]. One method, known as the Basic FANET Architecture, allows UAVs to communicate with each other and with the GS through a backbone UAV, as shown in Figure 3a. If there are multiple groups, this configuration is referred to as a Multi-Group FANET Architecture, illustrated in Figure 3b. As illustrated in Figure 3c, the configuration in which backbone UAVs connect to the GS (ground station) via other backbone UAVs, rather than connecting independently, is referred to as a Multi-Layer FANET Architecture.

In this paper, we present a novel authentication framework tailored for UAV systems using the capabilities of PUF-enabled authentication. Our approach aims to enhance the validity and security of communication protocols in practical settings, mitigating the risks posed by cyber threats. After a comprehensive review of the existing literature, we categorize relevant studies into detection and prevention techniques, providing insight into how UAV security is developing. Through outlining the critical need for strong security protocols, we highlight the necessity of mitigating escalating risks faced by UAVs, thereby ensuring their continued efficacy in a wide-range of applications [13].

The following sections explain the details of UAV cyber attacks, clarify the security goals of our suggested architecture, and outline the system model that supports our authentication method. Furthermore, we will elaborate on the practical implementation of PUF-enabled authentication and discuss potential areas for future research and innovation in UAV security.

The main contributions of this paper are as follows:

1. A novel mutual authentication protocol is introduced to enable a secure link between a UAV and the ground station utilizing Physical Unclonable Functions, without requiring any secret information to be stored on the UAV.
2. A comprehensive solution is provided for securing Flying Ad Hoc Networks. By integrating PUFs, the communication channels between UAVs and ground stations are secured against unauthorized access, and network resilience to various cyber threats is enhanced.
3. The proposed protocol is thoroughly analyzed regarding its security properties through formal verification methods and cryptanalysis. This detailed analysis demonstrates the protocol’s effectiveness in protecting UAV networks from a wide range of cyber attacks, setting a new benchmark for secure communication in UAV operations.

## 2. Related Work

Recent investigations have highlighted the potential of PUFs as a highly effective security measure for IoT (Internet of Things) and embedded systems applications [6,14,15,16]. Because of the natural variation derived during production, these devices give their outputs unique, non-replicable identities. Consequently, they are recognized not only as security roots but also as security primitives of hardware trust [17]. The operational principle of PUFs depends on a challenge–response mechanism, whereby a specific stimulus, or challenge, prompts a distinct output, or response, from the device. It is anticipated that even when identical manufacturing processes and designs are applied, two PUF devices will produce divergent responses to the same challenge. This unique characteristic of PUFs is leveraged in our protocol to generate a unique session key for each device equipped with a PUF, effectively providing each UAV in the network with a non-duplicable, PUF-based identifier. This is similar to a functional mechanism that inputs a challenge and yields a corresponding response. Hooper et al. have outlined a comprehensive defensive strategy against threats such as buffer overflows and Denial of Service (DoS) attacks targeting the Address Resolution Protocol (ARP) cache [18]. On the other hand, Blazy et al. have considered a sophisticated adversarial framework capable of executing fault injections, side-channel, and physical assaults on UAVs, utilizing a keyed hash function to derive subsequent keys from an initially stored key [19,20]. They offer a method of employing multiple key sequences to prevent attacks, although a breach obtaining a single key from each sequence could compromise the security of all subsequent communications. Abdallah et al. propose a basic security scheme for UAV surveillance networks that offers one-way but not mutual authentication [21]. Another study introduces a method for certificateless group key authentication tailored for non-secure UAV networks, yet this method is not considered lightweight due to its reliance on elliptical curve cryptography (ECC) and bilinear pairing [22]. Similarly, a strategy involving short certificate-based signature distribution is described, which, despite its complexity, employs public key cryptography techniques [23]. Further research has demonstrated the efficacy of PUFs in safeguarding wireless and unmanned IoT settings [24,25,26]. Chen and Willems propose a PUF-based secret key generation mechanism [27]. In another recent study focusing on UAV authentication using PUFs, the PUF’s challenge-response pair initiates a chaotic system, enabling the covert exchange of random seeds for session key generation [28].

In addition to these foundational works, recent advancements in PUF technology have introduced machine learning-resistant designs that incorporate extra randomness and obfuscation layers, making them more robust against modeling attacks [29,30]. Furthermore, enhanced fuzzy extractor methods have been proposed to reduce error rates and maintain stable key generation in resource-constrained environments [31]. These developments are particularly relevant for UAV applications where lightweight and reliable security measures are essential [32].

On the UAV-centric side, state-of-the-art protocols integrate PUFs with low-complexity cryptographic primitives, ensuring reduced computation overheads while preserving secure mutual authentication. Post-quantum security considerations have also been explored, merging PUF-based device fingerprinting with quantum-resistant key-exchange methods to future-proof UAV networks [33]. Such approaches not only mitigate traditional threats like GPS spoofing or buffer overflows, but also address more complex adversarial scenarios in UAV swarms, highlighting the growing importance of PUF-based frameworks in modern UAV security [34].

### Common UAV Cyber Attacks

In today’s interconnected world, UAVs are increasingly used for various critical applications, making them prime targets for cyber attacks. Understanding the common types of cyber attacks that UAVs may face is essential for developing robust security measures. These attacks can be categorized based on their impact on integrity, availability, confidentiality, and authentication. Below, we outline some of the most prevalent types of cyber attacks against UAVs.

Integrity attacks on UAV systems primarily involve the manipulation of data to deceive or mislead the UAV’s decision-making processes. One common form of this attack is GPS spoofing, where the GPS signals received by the UAV are altered to misrepresent its geographical location, leading the UAV astray. Another form is the dissemination of false information, where misleading data are fed to the UAV, causing it to make incorrect decisions [35]. Both types of attacks can severely compromise the integrity of UAV operations, leading to potentially dangerous consequences.

Availability attacks are aimed at disrupting the normal communication and operation of UAVs. Jamming attacks, for instance, involve deliberate interference with UAV communication channels, effectively disrupting the flow of data and rendering the UAV inoperative [36]. Similarly, gray-hole and black-hole attacks selectively drop or divert communication packets, which can interrupt critical data transmission and impair the UAV’s functionality. These attacks can prevent UAVs from completing their missions and disrupt their operations significantly.

Confidentiality attacks focus on unauthorized access to sensitive information transmitted between UAVs and ground stations. Eavesdropping is a prevalent attack where malicious actors intercept UAV communications to gather confidential data [37]. Man-in-the-middle (MITM) attacks are another severe threat, where attackers intercept and modify communication between UAVs and ground stations, potentially altering critical information. Replay attacks also pose a significant risk, as attackers capture and retransmit data packets to impersonate legitimate UAVs, leading to unauthorized access and control.

Authentication attacks target the identity verification mechanisms of UAV systems. Cloning attacks involve replicating UAV identifiers or authentication credentials to impersonate legitimate UAVs, gaining unauthorized access [38]. Masquerade attacks also deceive ground stations by impersonating legitimate UAVs, leading to unauthorized operations. Additionally, node tampering, which includes physical or electronic alteration of UAV components, can compromise the integrity and functionality of the UAV. These attacks undermine the trust and security of UAV operations, making robust authentication mechanisms essential.

**Figure 4:**
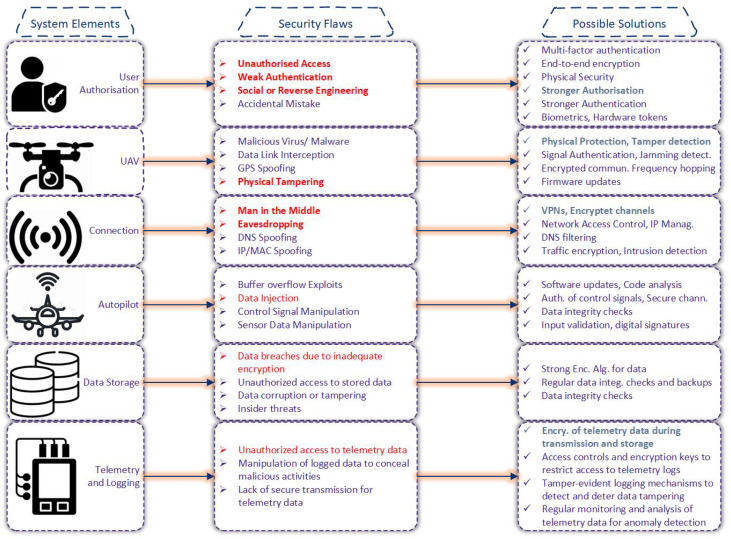
Security flaws in UAV system.

Figure 4 presents a detailed overview of security vulnerabilities within various UAV system elements and proposes potential solutions. The figure is structured into three columns: system elements, security flaws, and possible solutions. The system elements include User Authorization, UAV, Connection, Autopilot, Data Storage, and Telemetry and Logging. Each element is susceptible to specific cyber attacks; for instance, User Authorization can face unauthorized access and impersonation, while the UAV itself is vulnerable to physical tampering and firmware attacks. Connection issues like jamming and MITM attacks, Autopilot threats such as GPS spoofing and false command injections, Data Storage vulnerabilities involving unauthorized access, and Telemetry and Logging susceptibilities to data manipulation are all highlighted. To counter these threats, the figure suggests robust solutions such as multi-factor authentication, secure communication protocols, encryption, and secure logging mechanisms. By implementing these solutions, the security and reliability of UAV systems can be significantly enhanced, ensuring they are better protected against various cyber threats.

## 3. Security Objectives and System Model

### 3.1. Threat Model

The security of UAV communication channels in a FANET environment is crucial due to the highly distributed nature of UAV operations, the sensitive information they carry, and the vulnerability of wireless communication. Our threat model assumes a strong adversary with significant capabilities to compromise UAV communications. The model is based on the Dolev–Yao threat model and addresses a range of attack vectors, including communication-based threats and physical security risks [39].

- Eavesdropping: An Adv can intercept and listen to all unencrypted communications across the network. This allows the adversary to gather information about the communication protocol, message contents, and network topology.
- Message Modification and Injection: An Adv can intercept, delete, modify, and insert messages in public communication channels. This capability includes the ability to create spoofed messages to impersonate a legitimate UAV or ground station.
- Replay Attacks: An Adv can store intercepted messages and replay them at a later time, potentially bypassing nonces or other security mechanisms used to prevent duplication.
- Man-in-the-Middle Attacks: Advs can position themselves between two legitimate communication parties and alter the communication stream, potentially compromising the integrity and confidentiality of the data.
- Device Capture and Tampering: An Adv can physically capture a legitimate UAV and attempt to extract sensitive information from its memory, including encryption keys, mission data, and authentication credentials.
- Cloning Attack: After capturing a UAV, an Adv may attempt to create a clone of the device by extracting its memory contents, allowing the adversary to masquerade as the legitimate UAV.

### 3.2. Design Goals

The proposed protocol is designed to ensure secure communication between UAVs and the GS, addressing a range of critical security challenges. The primary goals of the design are as follows:

Mutual Authentication: The protocol should guarantee that only legitimate UAVs can successfully authenticate with the GS, ensuring protection against impersonation attacks. Both the UAV and the GS must prove their identities to each other to establish trust.

Session Key Agreement: The protocol must generate unique session keys for each authentication session to ensure secure data communication between UAVs and the GS, preventing unauthorized data interception.

Session Key Independence: Each session key should be independent from previous sessions, ensuring that compromising one session key does not affect the security of subsequent sessions.

Anonymity Protection: The protocol must preserve the anonymity of each legitimate UAV, preventing adversaries from identifying the real identity of UAVs by eavesdropping on communication channels.

Untraceability: The design should ensure that no unauthorized entity can trace the communication or the identity of UAVs, even if authentication messages are intercepted.

Resistance to Physical Attacks: The protocol must be resilient to physical attacks, such as UAV node capture or tampering, safeguarding against vulnerabilities in hardware-based attacks.

Clock Synchronization Independence: Given the challenges of wireless communication and potential clock drift in UAVs, the protocol should function without requiring precise synchronization between the UAV and GS clocks.

Forward Secrecy: The design must ensure perfect forward secrecy, meaning session keys remain secure even if long-term secrets are compromised.

Resistance to Known Attacks: The protocol must defend against a range of known malicious attacks, including eavesdropping, masquerading, MITM, replay attacks, node tampering, and cloning.

### 3.3. System Model

Our proposed system is designed to securely authenticate communications both between UAVs and a GS, and directly between UAVs themselves. The model leverages the robust security framework established in the UAV-to-GS authentication to facilitate secure UAV-to-UAV communications. This design ensures that UAVs authenticated through the GS can establish secure links with one another based on the trust model and security assurances provided by the GS. The system model is set within a Basic FANET Architecture, as illustrated in Figure 3a, where a backbone UAV facilitates communication. Before exploring the specifics of the proposed protocol, it is crucial to clarify the terminology used. Table 1 lists and defines these terms to enhance understanding and ensure clarity.

## 4. The Proposed Scheme

Our approach to securing UAV communications within FANET is structured into three distinct phases: UAV Registration, UAV-GS Authentication, and UAV-UAV Authentication. This structure is designed to robustly safeguard the identity and integrity of communications at every step, employing mechanisms such as disposable pseudonyms and masked identities to ensure confidentiality and prevent unauthorized access.

### 4.1. UAV Registration Stage

Before UAVs are deployed in the field, they must undergo a registration process with the ground station. This phase establishes a secure baseline for subsequent authentication and communication, providing critical data to identify and verify UAVs. The proposed authentication protocol leverages Physical Unclonable Functions to ensure secure UAV communication through a challenge–response mechanism. Each UAV is equipped with a unique PUF, which generates device-specific responses based on intrinsic physical variations in its hardware. During the registration phase, the ground station sends a predefined set of challenge inputs $C_{i}$ to the UAV. The UAV processes these challenges through its PUF, producing unique response outputs $R_{i}$ that are then securely stored at the GS. In the protocol, the UAV stores only the first challenge $C_{0}$ locally, while 12 challenge–response pairs (C-R) are registered in the ground station.

#### 4.1.1. Initial Registration

UAV Assignment: Each UAV *Ui* is assigned a unique permanent identity that will be used for internal tracking within the ground station’s database.

Challenge–Response Pair Generation: The ground station generates a series of random challenges (*Ci*) and sends them to the UAV. The UAV, using its Physical Unclonable Function (PUF), calculates the corresponding responses (*Ri*) and sends them back to the ground station.

#### 4.1.2. Data Storage and Handling

Challenge–Response Pair Storage: The ground station stores the list of challenge–response pairs in its database. This list will be used to authenticate the UAV during the UAV-GS authentication phase.

Temporary Identifier Assignment: Alongside the challenge–response pairs, the ground station assigns a temporary identifier (*tid*) to each UAV, which will be used during ongoing operations to maintain anonymity.

Data on UAV and GS: The ground station retains the UAV’s permanent identity $U_{i}$, the assigned temporary identifier $tid_{i}$, the challenge–response pair list $C_{i},R_{i}$, and the identifier of the ground station itself (*Gi*). Each UAV holds a subset of this information, including the first challenge–response pair $C_{0},R_{0}$, the temporary identifier (*tid*), the identifier of its ground station (*Gi*), and its permanent identity (*Ui*).

#### 4.1.3. Resilience Against Desynchronization Attacks

Backup Challenge–Response Pairs: To mitigate the risk of desynchronization attacks, where the stored data on the UAV and GS could become misaligned, multiple challenge–response pairs are kept in reserve. This redundancy allows for re-authentication if any inconsistencies occur.

Re-authentication Mechanism: If desynchronization occurs, the ground station can utilize the backup challenge–response pairs to re-establish a secure authentication process with the UAV. By following these steps, the registration phase establishes a secure foundation for the subsequent UAV-GS authentication phase, ensuring that each UAV can be uniquely and securely identified while allowing flexibility and redundancy in the case of desynchronization or other operational challenges.

### 4.2. UAV-GS Authentication Phase

**Figure 5:**
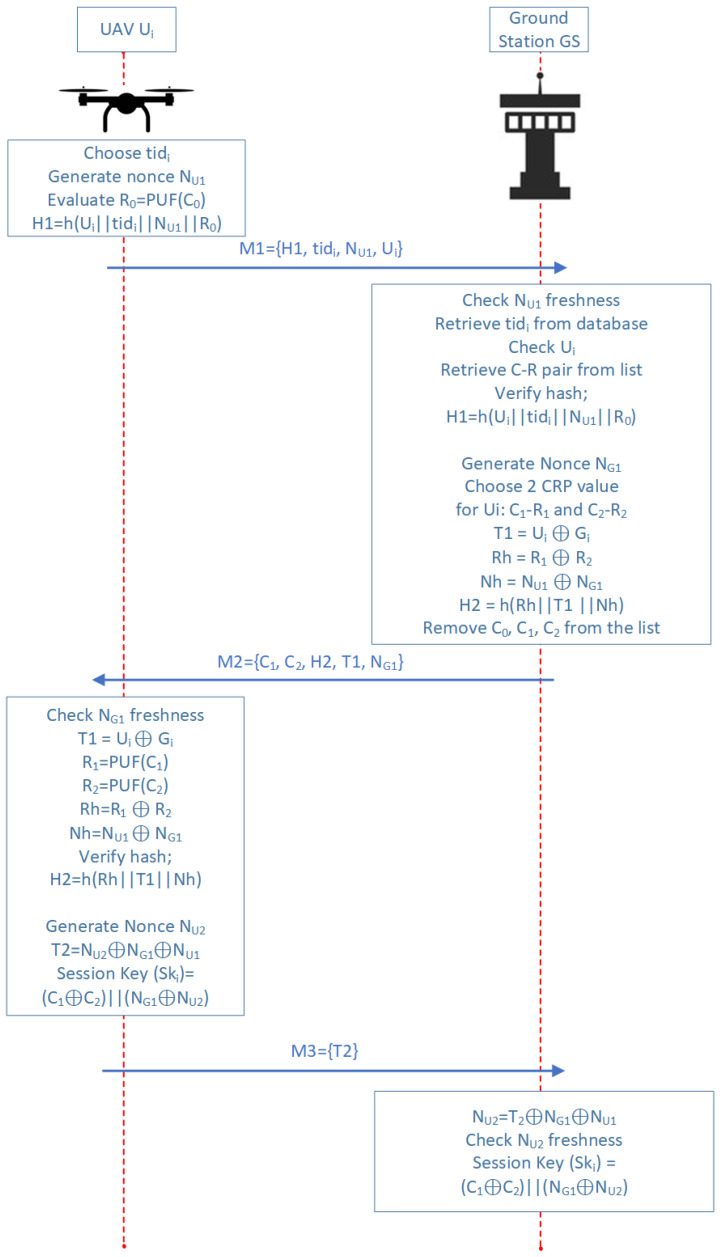
Authentication process between UAV and GS.

The authentication phase of the proposed scheme outlines the detailed process through which a UAV $U_{i}$ establishes its legitimacy to communicate with a ground station $G_{i}$. This phase focuses on key negotiations and the secure exchange of information between these two entities. Figure 5 summarizes the authentication process between the UAV and the GS. This figure illustrates the step-by-step communication and information exchange that occurs during the authorization phase to ensure secure and authenticated interactions. The primary goal of this phase is to ensure that only authenticated UAVs can initiate a secure communication session with the GS, preventing unauthorized access or malicious interference. During this phase, a secure session key is generated to ensure the confidentiality and integrity of subsequent communications between the UAV and the GS. This session key serves as a cornerstone for secure data transmission, providing the basis for encrypted communication that safeguards against eavesdropping and tampering. One of the key aspects of this authentication method is that it does not require UAVs to carry or store complex encryption keys, which would increase the risk of key exposure or compromise in the event of UAV capture or tampering. Instead, the UAV retains only a single challenge, which is used in the initial communication to initiate the authentication process. This simplification reduces the UAV’s memory and computational load while maintaining high security standards.

The UAV’s first message in the authentication process contains H1, which is derived from $R_{0}$. This message is verified by the GS to ensure the correctness of the initial step. The GS then selects two challenges, $C_{1}$ and $C_{2}$, from its list and sends them to the UAV. Upon receiving these challenges, the UAV uses its PUF to generate the corresponding $R_{1}$ and $R_{2}$ responses. The UAV then constructs a message using these responses, sending it back to the GS. The GS compares the UAV’s generated message with its own, verifying that the challenges and responses match. This no-password approach ensures that no sensitive information, such as static encryption keys, is ever exchanged or stored on the UAV.

By combining PUF-based authentication with dynamic session key generation, the system ensures robust protection against impersonation, cloning, and man-in-the-middle attacks. This makes it highly suitable for securing UAV networks in dynamic and adversarial environments, where UAVs are frequently deployed and operated under varying and potentially hostile conditions. The UAV does not store encryption keys, thus eliminating key storage vulnerabilities while still maintaining a secure communication channel between UAVs and the GS.

1. To send the initial authentication message, the UAV $U_{i}$ generates the first response $R_{0}$ by using the stored challenge $C_{0}$.
2. The UAV $U_{i}$ sends the message M1 = ($H1,tid_{i}$,$N_{U1}$) to the ground station. This message includes the temporary identifier ${tid}_{i}$, a random nonce $N_{U1}$, and a hash H1 = *h* ($U_{i}$‖${tid}_{i}$‖$N_{U1}$‖$R_{0}$), where $R_{0}$ is the response generated in step 1.
3. Upon receiving M1, the ground station checks its database for any entry corresponding to the received temporary id, ${tid}_{i}$. It also verifies the freshness of the nonce $N_{U1}$ to ensure it has not been used in previous authentication sessions. If these conditions are satisfied, $U_{i}$’s authentication request proceeds; if not, the request is disregarded. Following successful verification of the hash H1, the GS searches for the appropriate challenge–response pair (*C*,*R*) in its database. It then generates a new nonce $N_{G1}$ and selects two unused challenge–response pairs from the database for subsequent steps in the authentication process.
  (1) $$T1=U_{i}⊕G_{i},$$
  (2) $$Rh=R_{1}⊕R_{2},$$
  (3) $$Nh=N_{U1}⊕N_{G1},$$
  (4) H2=h(Rh∥T1∥Nh),
  (5) $$M2=(C_{1},C_{2},H2,T1,N_{G1}),$$
4. The GS then sends M2 = ($C_{1}$,$C_{2}$,H2,T1,$N_{G1}$) to $U_{i}$
5. The UAV $U_{i}$ checks the freshness of the incoming nonce $N_{G1}$ and retrieves the ground station’s identity ($G_{i}$) using T1 from the incoming message, along with its own identifier $U_{i}$.
  (6) $$G_{i}=U_{i}⊕T1,$$
  (7) $$R_{1}=PUF\left(C_{1}\right),$$
  (8) $$R_{2}=PUF\left(C_{2}\right),$$
  (9) $$R_{1}⊕R_{2}=Rh,$$
  (10) $$N_{U1}⊕N_{G1}=Nh,$$

The UAV $U_{i}$ uses challenges $C_{1}$ and $C_{2}$ as inputs to its PUF, generating responses $R_{1}$ and $R_{2}$. These responses are combined using a bitwise XOR operation to produce a composite response Rh. At the same time, $U_{i}$ calculates a new value, Nh by combining its original nonce $N_{U1}$ with the nonce $N_{G1}$ received from the ground station.

The UAV then checks the hash provided by the GS to ensure its integrity. If the hash fails to verify, the UAV terminates the authentication process, signaling a potential security issue. If the hash validation is successful, the UAV generates a new random nonce, $N_{U2}$. These new data are then used to encode T2, the next message in the communication sequence, as can be seen in (11).

(11) $$T2=N_{U2}⊕N_{G1}⊕N_{U1},$$

The calculation of the session key $Sk_{i}$, which is intended for use in later communication, is shown in (12).

(12) $$Sk_{i}=\left(C_{1}⊕C_{2}\right)∥\left(N_{G1}⊕N_{U2}\right),$$

6. The final message, M3={T2}, is transmitted by the UAV to the GS. Upon receiving the T2 message, the GS calculates $N_{U2}$ using the formula provided in (13) and checks its freshness. Subsequently, the session key $Sk_{i}$ is computed following the same method as in the UAV, as depicted in (12).
  (13) $$N_{U2}=T2⊕N_{G1}⊕N_{U1},$$
7. Consequently, the mutual authentication between the UAV and the GS is successfully completed. Following this, all subsequent communications are secured through encryption utilizing the session key Ski, which has been established to ensure data integrity and confidentiality.
8. As outlined in the equation below, to preserve user anonymity throughout the authentication process, both the UAV and the GS generate a new temporary identifier, ${\mathrm{tid}}_{i}^{\prime }$, for the UAV. This identifier will be used in future authentications to ensure anonymity.
  (14) $${{tid}_{i}}^{\prime }=H(N_{G1}∥{tid}_{i}∥Rh)\;\mathrm{mod}\;2^{64}$$

In this equation, $N_{G1}$ is a 160-bit random nonce generated by the ground station, $tid_{i}$ is the current temporary ID of the UAV $U_{i}$, and Rh is a 320-bit value. These three components are concatenated to form a 544-bit string. This string is then processed by a one-way hash function (SHA-1), yielding a 160-bit output. Finally, the $\;\mathrm{mod}\;2^{64}$ operation is applied to extract the last 64 bits, which become the new $tid_{i}^{\prime }$.

At the conclusion of this authentication protocol, both the UAV and the GS generate a highly secure session key. This session key is used to encrypt and secure all subsequent communications between the UAV and the GS, ensuring that the data transmitted remain confidential and protected from potential attacks. If the authenticated UAV lands and another UAV is deployed to take its place, a new authentication protocol must be executed. This process involves the newly deployed UAV undergoing the same rigorous authentication procedure with the GS to establish its identity and legitimacy. During this new authentication phase, a fresh session key is generated, specific to the new UAV and the GS, to ensure the continuity of secure communications. This approach not only maintains the security integrity of the network by preventing unauthorized access, but also ensures that each UAV operating within the FANET is individually authenticated and granted a unique session key. Such measures are crucial in dynamic and potentially hostile environments, where UAVs frequently enter and exit the network, thereby maintaining robust security and operational efficiency.

### 4.3. UAV-UAV Authentication Phase

**Figure 6:**
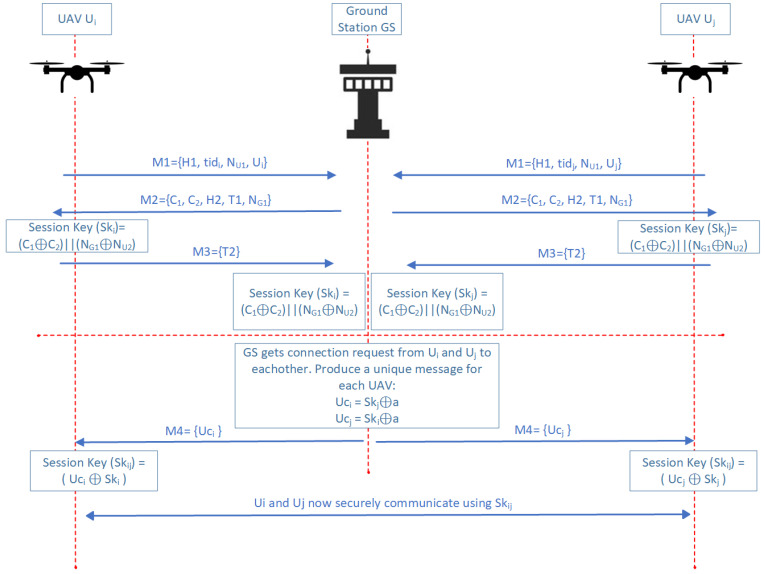
The authentication process between UAVs through the GS.

In the subsequent phase, we examine the authentication process between two UAVs, $U_{i}$ and $U_{j}$. Like the previous authentication phase, a series of messages exchanged in the protocol and the communication between the UAVs and the ground station are depicted in Figure 6. For simplicity, we assume that $U_{i}$ intends to initiate authentication with UAV $U_{j}$. Once a session key has been established between $U_{i}$ and the ground station GS, and between $U_{j}$ and GS, GS generates a unique message for $U_{i}$ which is $U_{ci}$ and for $U_{j}$ which is $U_{cj}$. GS generates $U_{ci}$ and $U_{cj}$ by XORing a randomly generated 192-bit number *a* with each UAV’s session key. This step ensures that the M4 message sent to the UAVs remains secure against potential eavesdropping attacks. Then each UAV produces a session key $SK_{ij}$ by performing an XOR operation on the last incoming message from the GS and their previous session key between them and the GS. This session key is used to facilitate secure communication between the parties.

The steps involved in the UAV-UAV authentication phase can be outlined as follows:

1. **Session Key Establishment:**
  - $U_{i}$ establishes a session key $sk_{i}$ with GS.
  - $U_{j}$ establishes a session key $sk_{j}$ with GS.
2. **Generation of Unique Message for UAVs:**
  - GS computes the $U_{ci}$ and $U_{cj}$ messages for UAVs.
    (15) $$Uc_{i}=Sk_{j}⊕a$$
    (16) $$Uc_{j}=Sk_{i}⊕a$$
3. **Distribution of Shared Session Key:**
  - GS securely transmits $Uc_{i}$ to $U_{i}$.
  - GS securely transmits $Uc_{j}$ to $U_{j}$.
4. **Secure Communication:**
  - $U_{i}$ and $U_{j}$ obtain $Sk_{ij}$ for their secure communication.
    (17) $$Sk_{ij}=Uc_{i}⊕Sk_{i}$$
    (18) $$Sk_{ij}=Uc_{j}⊕Sk_{j}$$

This phase ensures that both UAVs can authenticate each other and establish a secure communication channel facilitated by the ground station GS, leveraging the session keys established in their respective authentication phases with GS.

## 5. Security Analysis

In this section, we conduct a thorough security analysis of the UAV-GS authentication protocol. The analysis is divided into two parts: a formal analysis and a cryptanalysis. The formal analysis uses Mao and Boyd’s logic to rigorously prove the security properties of the protocol. The cryptanalysis is performed to examine the protocol’s resistance to various types of attacks and vulnerabilities.

### 5.1. Formal Proof

The formal analysis aims to establish the security properties of the UAV-GS authentication protocol using Mao and Boyd’s logic [40]. This formalism allows us to methodically verify authentication, nonce verification, confidentiality, super-principal, freshness, and good-key properties. By applying these logical rules, we can ensure that the protocol is robust against various security threats.

Before presenting the formal proofs, we briefly clarify the notation, parameters, and functions used throughout:

#(*X*) Represents the belief that *X* is fresh.
*A*|~*P* “Believes” operator—if A∣∼P, then *A* believes the proposition *P* is true.
*A*|⇒*M* “Sends” operator—if A∣⇒M, then *A* sends message *M*.
*K*(*A*,*B*) Symbol that *A* and *B* share a secure key (or that *A* believes the key is known only to *A* and *B*).

#### 5.1.1. Authentication Rule

**Goal:** The GS believes that the UAV is authenticated.

1. The GS receives message M1:
  $$GS|\Rightarrow \{H1,tid_{i},N_{U1},U_{i}\}$$
2. The GS checks the freshness of $N_{U1}$:
  $$GS|∼\#(N_{U1})$$
3. The GS verifies the hash H1 using $R_{0}$:
  $$GS|∼h(U_{i}∥tid_{i}∥N_{U1}∥R_{0})=H1$$
4. The GS sends message M2:
  $$GS|\Rightarrow \{C_{1},C_{2},H2,T1,N_{G1}\}$$
5. The UAV receives message M2:
  $$UAV|\Rightarrow \{C_{1},C_{2},H2,T1,N_{G1}\}$$
6. The UAV checks the freshness of $N_{G1}$:
  $$UAV|∼\#(N_{G1})$$
7. The UAV verifies the hash H2:
  UAV∣∼h(Rh∥T1∥Nh)=H2

##### Applying Authentication Rule


$$\frac{UAV|∼\#\left(N_{U1}\right)\;UAV|∼GS|∼h(U_{i}∥tid_{i}∥N_{U1}∥R_{0})}{UAV|∼GS|∼h(U_{i}∥tid_{i}∥N_{U1}∥R_{0})}$$

$$\frac{GS|∼\#\left(N_{G1}\right)\;GS|∼UAV|∼h\left(Rh∥T1∥Nh\right)}{GS|∼UAV|∼h(Rh∥T1∥Nh)}$$

#### 5.1.2. Nonce Verification Rule

**Goal:** Both the UAV and GS confirm the freshness of nonces.

1. The UAV generates $N_{U1}$:
  $$UAV|∼\#(N_{U1})$$
2. The GS checks $N_{U1}$ freshness:
  $$GS|∼\#(N_{U1})$$
3. The GS generates $N_{G1}$:
  $$GS|∼\#(N_{G1})$$
4. The UAV checks $N_{G1}$ freshness:
  $$UAV|∼\#(N_{G1})$$

##### Applying Nonce Verification Rule


$$\frac{GS|∼\#\left(N_{U1}\right)\;GS|∼UAV|∼h(U_{i}∥tid_{i}∥N_{U1}∥R_{0})}{GS|∼UAV|∼h(U_{i}∥tid_{i}∥N_{U1}∥R_{0})}$$

$$\frac{UAV|∼\#\left(N_{G1}\right)\;UAV|∼GS|∼h\left(Rh∥T1∥Nh\right)}{UAV|∼GS|∼h(Rh∥T1∥Nh)}$$

#### 5.1.3. Confidentiality Rule

**Goal:** The session key $Sk_{i}$ is known only to the UAV and GS.

1. The UAV calculates $Sk_{i}$:
  $$UAV|∼Sk_{i}=\left(C_{1}⊕C_{2}\right)∥\left(N_{G1}⊕N_{U2}\right)$$
2. The UAV sends T2 to the GS:
  UAV∣⇒{T2}
3. The GS calculates the same $Sk_{i}$:
  $$GS|∼Sk_{i}=\left(C_{1}⊕C_{2}\right)∥\left(N_{G1}⊕N_{U2}\right)$$

Since the key derivation is based on secret components and fresh nonces, neither party believes an adversary can reconstruct $Sk_{i}$.

#### 5.1.4. Super-Principal Rule

**Goal:** Both the UAV and GS trust the same session key.

1. The UAV believes that the session key $Sk_{i}$ is secure between the UAV and GS:
  UAV∣∼K(UAV,GS)
2. The GS believes that the session key $Sk_{i}$ is secure between the UAV and GS:
  GS∣∼K(UAV,GS)

#### 5.1.5. Freshness Rule

**Goal:** The session key $Sk_{i}$ is fresh.

1. The UAV generates nonce $N_{U1}$:
  UAV∣∼#(Nu1)
2. The GS checks the freshness of $N_{U1}$:
  $$GS|∼\#(N_{U1})$$
3. The GS generates nonce $N_{G1}$:
  $$GS|∼\#(N_{G1})$$
4. The UAV checks the freshness of $N_{G1}$:
  $$UAV|∼\#(N_{G1})$$

##### Applying Freshness Rule


$$\frac{UAV|∼\#\left(N_{U1}\right)\;GS|∼\#\left(N_{G1}\right)}{UAV|∼\#(Sk_{i})}$$

#### 5.1.6. Good-Key Rule

**Goal:** $Sk_{i}$ is a good key for secure communication.

1. The UAV believes that the session key $Sk_{i}$ is secure between the UAV and GS:
  UAV∣∼K(UAV,GS)
2. The GS believes that the session key Ski is secure between the UAV and GS:
  GS∣∼K(UAV,GS)

Hence, both parties are convinced of the key’s security and suitability for communication.

**Figure 7:**
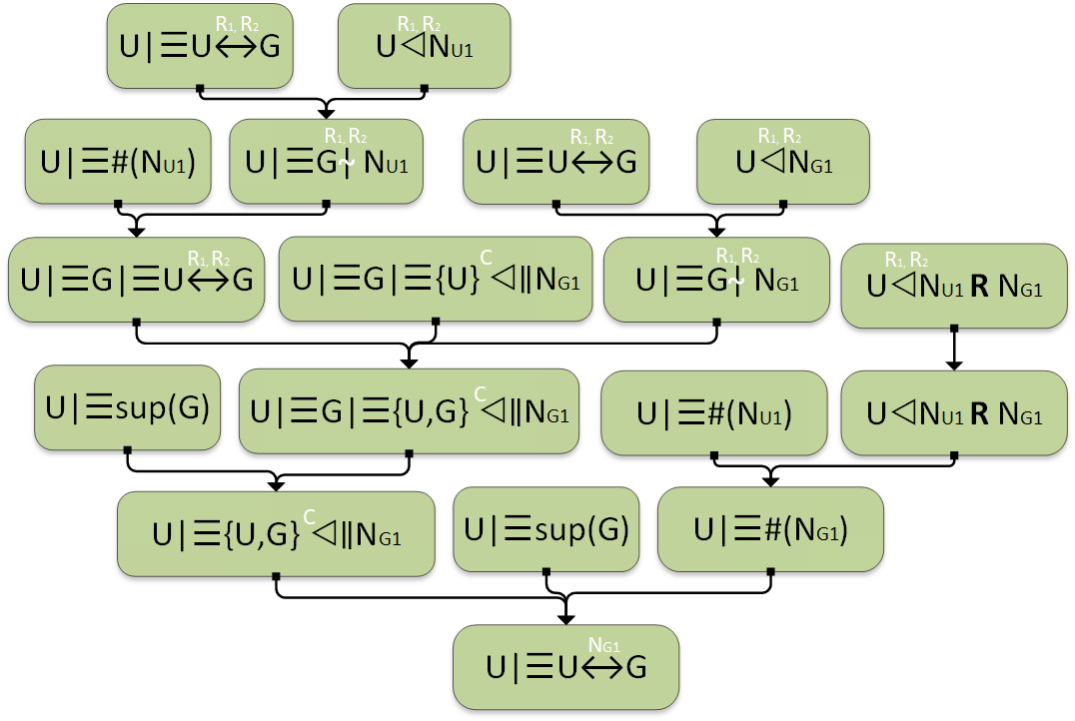
Logical proof of the protocol.

To validate the statement that “U believes $N_{G1}$ is a secure secret between U and G”, we provide a proof using Mao and Boyd’s logic. The proof is structured through Equations (19)–(33), with a corresponding visual representation illustrated in Figure 7. This demonstration establishes the logical foundation for the claim, reinforcing the notion that U considers $N_{G1}$ to be a securely shared secret between U and G.

The responses $R_{1}$ and $R_{2}$, which correspond to the challenge–response pairs ($C_{1}$, $R_{1}$) and ($C_{2}$, $R_{2}$) of U, are stored in G. As a result, “U believes $R_{1}$ and $R_{2}$ are securely shared secrets between U and G” (19). Utilizing $R_{1}$ and $R_{2}$, the UAV U can decrypt Rh and H2 from the second protocol message M2 = $C_{1}$, $C_{2}$, H2, T1, $N_{G1}$ to retrieve $N_{U1}$ and $N_{G1}$. Consequently, “U extracts $N_{U1}$ using the decryption keys $R_{1}$ and $R_{2}$” (20) and “U extracts $N_{G1}$ using the decryption keys $R_{1}$ and $R_{2}$” (21).

(19) $$U|\equiv U\overset{R_{1},R_{2}}{↔}G$$

(20) $$U\overset{R_{1},R_{2}}{◃}N_{U1}$$

(21) $$U\overset{R_{1},R_{2}}{◃}N_{G1}$$

By applying the authentication rule to statements (19) and (20), we derive that “U believes G encrypted $N_{U1}$ using $R_{1}$ and $R_{2}$” (22). Likewise, using the same rule on statements (19) and (21), we obtain “U believes G encrypted $N_{G1}$ using $R_{1}$ and $R_{2}$” (23). Given that U generates a new nonce NG for each session, it follows that “U believes $N_{U1}$ is fresh” (24).

Next, applying the nonce verification rule to statements (22) and (24) leads to the conclusion that “U believes G considers $R_{1}$ and $R_{2}$ to be a secure secret between U and G” (25). This logical deduction reinforces U’s belief that G acknowledges $R_{1}$ and $R_{2}$ as a confidentially shared secret within their communication.

(22) $$U|\equiv G\overset{R_{1},R_{2}}{|∼}N_{U1}$$

(23) $$U|\equiv G\overset{R_{1},R_{2}}{|∼}N_{G1}$$

(24) $$U|\equiv \#(N_{U1})$$

(25) $$U|\equiv G|\equiv U\overset{R_{1},R_{2}}{↔}G$$

Since G generates a new nonce NG with each iteration, U understands that $N_{G1}$ remains unknown to any entity other than G. Consequently, U believes that “G considers $N_{G1}$ inaccessible to anyone except U” (26). Applying the confidentiality rule to statements (23), (25), and (26), we derive that “U believes G is certain that no entity other than U and G has access to $N_{G1}$” (27). This conclusion reinforces the notion that only U and G possess authorized access to $N_{G1}$, aligning with U’s belief and G’s acknowledgment of its exclusivity.

(26) $$U|\equiv G|\equiv \left\{U\right\}\overset{C}{◃}\|N_{G1}$$

(27) $$U|\equiv G|\equiv \left\{U,G\right\}\overset{C}{◃}\|N_{G1}$$

The protocol operates under the assumption that U trusts the ground station G as a secure and reliable entity, meaning “U believes G holds the role of a super-principal” (28). By applying the super-principal rule to statements (27) and (28), it follows that “U believes $N_{G1}$ is inaccessible to anyone except U and G” (29). This affirms that U is confident that only U and G have the necessary access rights to $N_{G1}$, reinforcing the protocol’s security and the confidentiality of their exchanged information.

(28) U∣≡sup(G)

(29) $$U|\equiv \left\{U,G\right\}\overset{C}{◃}\|N_{G1}$$

$N_{U1}$ can be identified as the challenge, while $N_{G1}$ serves as the response. It is essential to distinguish $N_{U1}$ and $N_{G1}$ from the PUF-based challenge–response pair (C, R). As a result, the statement “U can observe the replied challenge $N_{U1}$ and the response $N_{G1}$ using the decryption keys $R_{1}$ and $R_{2}$” (30) holds. Applying the intuitive rule to statement (30) allows us to simplify it to “U can observe the replied challenge $N_{U1}$ and the response $N_{G1}$” (31).

(30) $$U\overset{R_{1},R_{2}}{◃}N_{U1}RN_{G1}$$

(31) $$U◃N_{U1}RN_{G1}$$

By applying the freshness rule to statements (24) and (31), we derive that “U believes $N_{G1}$ is fresh” (32). Subsequently, using the good-key rule on statements (28), (29), and (32), we establish that “U believes $N_{G1}$ is a securely shared secret known only to U and G” (33). This logical deduction reinforces U’s confidence that $N_{G1}$ remains confidential and exclusively accessible to U and G.

(32) $$U|\equiv \#(N_{G1})$$

(33) $$U|\equiv U\overset{N_{G1}}{↔}G$$

In conclusion, it has been established that the statement “G believes $N_{G1}$ is a securely shared secret known only to U and G” can be proven through a similar logical approach.

Additionally, the same reasoning can be applied to demonstrate that adversaries cannot obtain $N_{U2}$, $R_{1}$, or $R_{2}$. The confidentiality of $N_{G1}$, $N_{U2}$, $R_{1}$, and $R_{2}$ remains intact, regardless of the attack type—whether it be a masquerade attack, a man-in-the-middle attack, or a replay attack. Consequently, the exchanged data remain secure, ensuring that adversaries cannot intercept or decipher them.

By applying Mao and Boyd’s logic to the protocol, we demonstrate the following:

- **Authentication:** Both UAV and GS authenticate each other using fresh nonces and challenge–response pairs.
- **Nonce Verification:** Both nonces $N_{U1}$ and $N_{G1}$ are verified to be fresh, ensuring no replay attacks.
- **Confidentiality:** The session key $Sk_{i}$ is established securely, known only to the UAV and GS.
- **Super-Principal Rule:** The GS is trusted by the UAV to maintain secure challenge–response pairs and identities.
- **Freshness Rule:** The freshness of nonces ensures the authenticity and timeliness of the messages.
- **Good-Key Rule:** The session key $Sk_{i}$ is a good key for secure communication between the UAV and GS.

Thus, mutual authentication and secure communication are achieved in the protocol, ensuring the UAV and GS can communicate securely.

### 5.2. Cryptanalysis

This evaluation follows the security criteria list provided by the authors in [41], which addresses and removes uncertainties and redundancies commonly found in security protocols. In our analysis, we have excluded the following criteria as they are related to smart cards and user-input passwords, which are not relevant to our protocol: C1 (nNo password verifier-table), C2 (password friendly), C3 (no password exposure), C4 (no smart card loss attack), C6 (sound repairability), and C9 (timely typo detection). Several studies, such as [42,43], have adopted this advanced security evaluation method. In this cryptanalysis, we denote the GS as G, the UAV as U, and the adversary as A.

[C5] Resistance to known attacks:

The proposed scheme is resistant to MITM attacks (C5.1), masquerade attacks (C5.2), replay attacks (C5.3), node tampering attacks (C5.4), cloning attacks (C5.5), and desynchronization attacks (C5.6). To prevent MITM attacks, (C, R) pairs registered in G during registration are used only once. An adversary intercepting communication between U and G might capture a challenge–response pair from a previous authentication. However, G will not reuse the same challenge in subsequent authentications, thus preventing the adversary from replaying the response to G without U’s knowledge. Using a new pair from the CRP (challenge–response pair) list in each session secures the protocol against MITM attacks. The adversary A cannot impersonate a UAV due to the unique PUF, nor can it impersonate G since it lacks the correct (C, R) pairs. The protocol ensures that replay attempts by A fail because a new (C, R) pair is used in each session. Since PUFs cannot be cloned, A cannot successfully clone U, making the scheme secure against cloning attacks. Even if A captures U and attempts to tamper with the PUF, the PUF will become unusable, preventing node tampering attacks. Additionally, no critical authentication information is stored in U.

[C7] Provision of key agreement:

As demonstrated in the formal proof section, the secrecy of nonces Nu1, Ng1, and Nu2 is established. The value “Rh” is generated by XORing two distinct responses. Each session uses a new (C, R) pair, ensuring different R values for each session. The protocol establishes a new and secure session key $Sk_{i}=\left(C_{1}⊕C_{2}\right)∥\left(N_{G1}⊕N_{U2}\right)$ after successful authentication.

[C8] No clock synchronization:

Our protocol ensures message freshness using random nonces instead of timestamps. This eliminates the need for clock synchronization between the GS and UAV, thus avoiding issues related to time delays and clock synchronization.

[C10] Mutual authentication:

In the proposed scheme, a single PUF pair (C, R) of the UAV is stored on the GS before deployment. This setup allows a session key to be established only between the UAV and GS. Therefore, the establishment of a session key $Sk_{i}$ indicates mutual authentication and confirms the legitimacy of the communicating parties.

[C11] User anonymity:

The protocol ensures user anonymity by updating the temporary ID of $U_{i}$ after each authentication using the formula ${\mathrm{tid}\text{’}}_{i}=h(N_{G1}∥tid_{i}∥Rh)$ at the end of the process. Both $U_{i}$ and the GS can independently compute the same temporary ID without exchanging messages. For a given challenge *C*, only $U_{i}$ and the GS, who know the PUF response *R*, can determine the temporary ID.

[C12] Forward secrecy:

The proposed scheme guarantees that the security of future sessions remains intact even if A guesses the current session key Ski. This is because A cannot obtain the response for the next session, *R*, which is required to generate the session key for the next session, as established in the formal proof section. The protocol’s assurance of user anonymity (C11) also prevents A from tracking the UAV, further safeguarding future sessions. Consequently, the protocol ensures perfect forward secrecy. Additionally, A will not be able to obtain the session key of any previous session, as it cannot gather the necessary secrets. Thus, the protocol also guarantees perfect backward secrecy.

#### Attack Scenarios

Replay Attack:

Example: An adversary intercepts a valid (C, R) pair from a previous session and attempts to replay it in a new session.

Defense: Due to the protocol’s design, each session utilizes fresh nonces and a unique (C, R) pair. The protocol shows that the probability of a successful replay attack is negligible, as *G* verifies the freshness of nonces ($N_{G1}$, $N_{U1}$, and $N_{U2}$) before accepting any authentication attempt.

Cloning Attack:

Example: An adversary attempts to clone the UAV by capturing its PUF response.

Defense: The unique and unclonable nature of PUF responses ensures that even if an adversary captures the output, it cannot replicate it for future sessions. The inherent randomness and sensitivity of the PUF prevent successful cloning, thereby preserving the integrity of the UAV’s identity.

## 6. Results and Discussion

### 6.1. Security Comparison

In this section, we evaluate our protocol against several other UAV-GS authentication systems, focusing on communication cost and security aspects as the primary criteria. The comparative analysis is based on findings from the publications [14,28,44,45,46].

Table 2 presents a comparative analysis of the security features provided by various authentication protocols, including our proposed method. The proposed protocol has been rigorously tested for reliability against cyber attacks, as detailed in Section 5.1 and Section 5.2. As illustrated, our protocol matches the security capabilities of the compared works, offering comprehensive protection against threats including mutual authentication failures, replay attacks, message integrity breaches, tampering attacks, and MITM attacks. Notably, our protocol is validated through formal proof, further ensuring its robustness against these security challenges. Moreover, unlike some existing methods, our approach also includes identity protection, enhancing the overall security posture. Although two of the compared studies have provided sufficient protection against the mentioned threats, the proposed approach is prominent in terms of computation and communication costs. In particular, our protection method ensured an 18 percent lower communication cost compared with the best protocol with which it was compared in this study context. This parity in security features, combined with the optimizations discussed in Section 6.2, particularly in terms of communication cost, underscores the effectiveness of our protocol. It achieves a careful balance between high security standards and operational cost, making it a strong candidate for secure and efficient UAV communication networks.

To emulate the use of PUF in the authentication phase, we set up virtual server and client containers using Docker. The server container simulated the GS, while the client container represented the UAV. The process begins with the UAV initiating an authentication request to the GS. The GS then selects a challenge–response pair from its stored database and sends the challenge to the UAV. Upon receiving the challenge, the UAV uses the PUF mechanism to generate a response and sends this response back to the GS. The GS verifies the response; if the verification is successful, the GS sends a confirmation message to the UAV, indicating successful authentication. This setup effectively simulates the interaction between the GS and UAV, demonstrating the communication flow and successful authentication process using the PUF-based challenge–response mechanism.

Overall, the proposed protocol not only provides enhanced security against various cyber threats, but also optimizes communication resources. This makes it an ideal choice for modern UAV-GS authentication systems, where both security and efficiency are paramount.

### 6.2. Performance Comparison

In this section, we assess the communication cost of the proposed authentication scheme and compare it with the protocols presented in [14,28,44,45,46]. For a fair comparison, we standardized the bit sizes for UAV identification, nonces, and hash outputs at 160 bits each, while the lengths for temporary request identification and GS identification are set at 64 bits each. For our protocol, we utilize a recent PUF proposed in [47] to be deployed in the UAVs. This particular PUF has been shown to generate a response of at least 256 bits, with a 320-bit response produced in 0.4 µs when provided with a 32-bit challenge input. Additionally, the SHA-1 (Secure Hash Algorithm) processes inputs and generates a 160-bit hash value.

**Figure 8:**
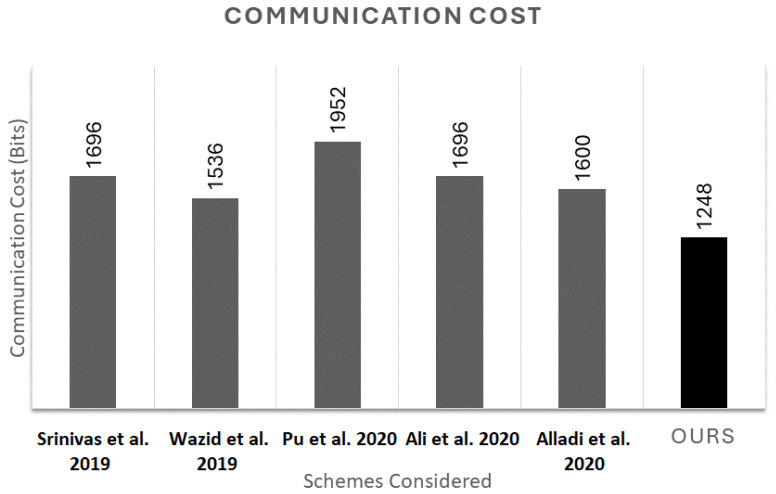
Comparison of communication costs in similar protocols [14,28,44,45,46].

In our proposed scheme, the lengths of messages M1, M2, and M3 are as follows: M1 has a length of (160 + 64 + 160 + 160) = 544 bits, M2 is (32 + 32 + 160 + 160 + 160) = 544 bits, and M3 is 160 bits. This results in a total communication overhead of 1248 bits between the UAV and the GS. As depicted in Figure 8, the total communication overheads for the schemes in [14,28,44,45,46] are 1696 bits, 1536 bits, 1952 bits, 1696 bits, and 1600 bits, respectively.

Our scheme demonstrates a significantly lower communication cost compared to these alternatives. This efficiency in communication is crucial in UAV systems, where minimizing communication overheads directly translates to reduced energy consumption and prolonged operational lifespan for the UAVs. Lower communication costs are particularly important in resource-constrained environments, such as UAV operations, where battery life and processing power are limited.

Moreover, our protocol maintains robust security features comparable to those provided by many established works in the literature. This balance of security and efficiency makes our protocol a highly effective solution for modern UAV networks. It ensures secure communication while optimizing the use of limited resources, thereby enhancing the overall performance and sustainability of UAV operations.

As UAV networks expand, ensuring scalability becomes increasingly important. While the proposed authentication protocol effectively balances security and efficiency, its ability to handle a growing number of UAVs must also be considered. Scalability is a crucial factor in maintaining network performance, as increasing UAV density can introduce authentication delays and computational overheads.

The current authentication mechanism relies on UAV-GS interactions using PUF-based challenge–response pairs. However, as UAV density grows, the GS must process an increasing number of authentication requests, which can lead to network congestion and higher latency in large-scale UAV networks. Implementing hierarchical or parallel authentication mechanisms can mitigate these delays and reduce network congestion.

Additionally, the GS is responsible for verifying each UAV’s authentication request, which demands computational power and storage. A significant increase in UAVs may result in higher energy consumption and processing delays. Furthermore, the GS must maintain a large database of CRPs for all registered UAVs, and as the network scales, storing and retrieving CRPs could introduce latency and require significant memory resources. Optimizing CRP management through dynamic challenge generation or distributed authentication methods can enhance scalability and system performance.

Beyond the reduction in communication cost, it is important to consider its real-world implications, particularly in terms of UAV battery life, mission duration, authentication speed, network congestion, and security. Communication modules are among the primary energy consumers in UAVs, and reducing the overhead associated with authentication directly decreases power consumption. In mission-critical applications such as disaster response, reconnaissance, and environmental monitoring, increased operational time enables UAVs to cover larger areas and reduces the frequency of battery recharges or replacements. Additionally, the optimized authentication process results in faster authentication times, ensuring UAVs can securely join the network without unnecessary delays. The lower bandwidth usage also helps minimize network congestion, improving real-time data exchange and overall system responsiveness, particularly in UAV swarms where multiple devices communicate simultaneously. Furthermore, a reduced communication overhead lowers the risk of cyber threats, as it decreases the exposure of authentication messages to potential interception or replay attacks. In dynamic environments where UAVs frequently join and leave the network, the improved efficiency ensures sustained authentication without compromising security. While these benefits highlight the practical impact of the proposed approach, future research could incorporate power consumption models in real-world UAV simulations to further quantify the relationship between authentication efficiency and operational longevity.

## 7. Conclusions

Our proposed authentication protocol for UAV networks enhances security while optimizing communication costs. By integrating Physical Unclonable Functions, hash functions, and session key agreements, the scheme provides strong protection against cyber threats with minimal overheads.

The reduced communication cost improves scalability and allows UAVs to operate efficiently, supporting applications such as disaster response, surveillance, and logistics. As UAV networks expand, our protocol remains a robust and practical solution for secure operations.

Despite its advantages, the protocol’s reliance on PUFs introduces sensitivity to environmental factors, potentially affecting reliability. However, incorporating error correction algorithms can improve response accuracy under varying conditions. Scalability challenges may arise in large UAV networks, particularly in managing session keys and cryptographic operations. Ensuring the system’s scalability is critical for FANET, requiring a re-evaluation of each protocol step to optimize performance at scale. Additionally, dependence on the ground station for key distribution could pose vulnerabilities in UAV-to-UAV communications. A head UAV-based authentication model could be explored as an alternative to a GS-centric approach, distributing authentication tasks more efficiently.

Future work will address these limitations by enhancing PUF reliability, exploring scalable cryptographic techniques, and testing real-world applications to refine security and efficiency further.

## Figures and Tables

**Table 1:** Notations used in this paper.

Notation	Meaning
U_*i*_	ID of UAV
Tid_*i*_	Temporary ID of UAV
GS	Ground station
G_*i*_	ID of ground station
N_*Ui*_	Random nonce produced by UAV
N_*Gi*_	Random nonce produced by GS
C_*i*_	$i^{th}$ challenge parameter of PUF
R_*i*_	$i^{th}$ response parameter of PUF
h()	Hash function
⊕	XOR operation
∥	Concatenation
Sk_*i*_	Session key between UAV and GS
Sk_*ij*_	Session key between $U_{i}$ and $U_{j}$
a	192-bit random number

**Table 2:** Comparison of security features.

Features	[14]	[44]	[46]	[28]	[45]	Ours
Mutual Authentication	✓	✓	✓	✓	✓	✓
Replay Attack	✓	✓	✓	✓	✓	✓
Message Integrity	✓	✓	✓	✓	✓	✓
Tampering Attack	✓	✓	✓	✓	X	✓
MITM	✓	✓	✓	✓	✓	✓
Formal Proof	✓	✓	✓	✓	✓	✓
Identity Protection	✓	✓	X	X	✓	✓

## Data Availability

Data are contained within the article.

## Abbreviations

The following abbreviations are used in this manuscript:

PUF	Physical Unclonable Function
UAV	Unmanned Aerial Vehicle
GS	Ground station
FANET	Flying Ad Hoc Network
VANET	Vehicular Ad Hoc Network
